# The role of pH in corrosion inhibition of tin using the proline amino acid: theoretical and experimental investigations†

**DOI:** 10.1039/d0ra04333h

**Published:** 2020-08-11

**Authors:** Brahim EL Ibrahimi, Lahcen Bazzi, Souad EL Issami

**Affiliations:** Applied Chemistry-Physic Team, Faculty of Sciences, University of Ibn Zohr P. O. Box 8106, Cité Dakhla Agadir Morocco brahimmhm@gmail.com +212672254020; Industrial & Logistic Laboratory, Higher School of Management, Telecommunications and Computer Science, SUP MTI Rabat Morocco

## Abstract

Herein, the effect of a mediums' pH on the interfacial interactions between proline (Pro) amino acid and tin metal was studied. In this work, acidic and near-neutral pH values were considered. DFT-based calculations and Monte Carlo simulations were carried out in the aqueous phase to understand the role of pH in Pro/tin interactions. To confirm this role in terms of inhibition efficiency, the CC and AC electrochemical techniques were used. Based on the calculated parameters, it was outlined that the partially protonated Pro form exhibits significant affinity to interact with tin metal in near-neutral media. However, in acidic media, its affinity is decreased, whereas the full protonated form was found to be solvated in the solution and its interaction with the tin surface was not favorable. These findings indicated that the inhibition capability of Pro for tin could be better in near-neutral media than in acidic media. This expected behavior was confirmed experimentally, wherein an inhibition effectiveness of around 65% was obtained for Pro at pH 5, and 16% was observed at pH 2. Additionally, the results highlight the capability of employing computational studies to predict the prevention efficiency of anti-corrosion compounds by varying the solution pH.

## Abstract 2

Herein, the effect of a mediums' pH on the interfacial interactions between proline (Pro) amino acid and tin metal was studied.

## Graphical abstract

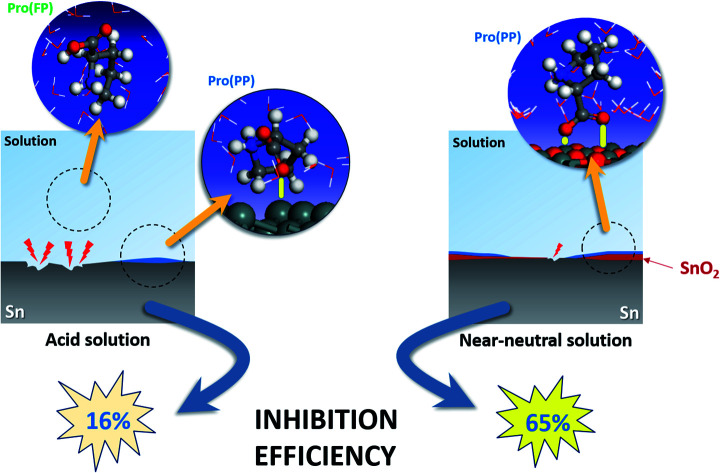

## 1 Introduction

**Fig. 1:**
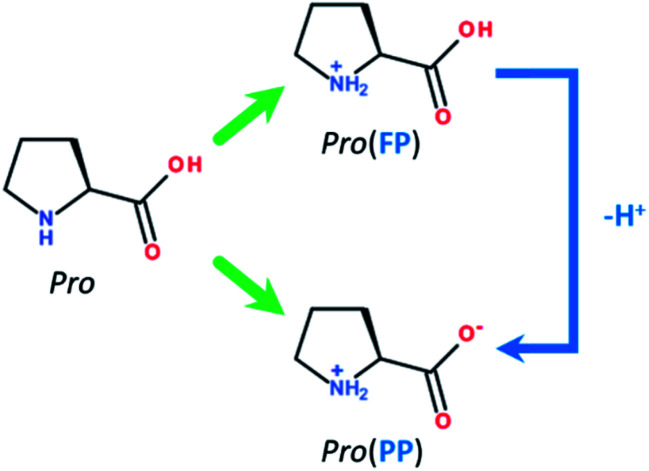
Molecular structures of Pro amino acid and its FP and PP forms.

Among the numerous available applications of tin, is its use as an internal metallic coat in diverse packaging.^1^ However, similar to most metallic materials, its contact with corrosive environments, such as saline and acid media, results in corrosion.^2^ Hence, the prevention of tin corrosion in these media has attracted industrial and academic attention. Accordingly, the use of corrosion inhibitor compounds is practical and an attractive method.^3^ To date, several organic molecules have been used as corrosion inhibitors, and among them, amino acids have been broadly investigated as eco-friendly and cheap anti-corrosion compounds for different metallic materials in various corrosive environments.^4–10^ In addition to the ecological advantage of amino acids, they are characterized by the presence of different heteroatoms (*e.g.*, O, N and S) and eventual unsaturated bonds within their molecular skeletons.^11,12^ These molecular features can effectively facilitate the adsorption of amino acids onto the surface of metals and result in the inhibition of metal corrosion.^13^ According to the available literature, cysteine and methionine amino acids have been studied extensively.^14–20^ Nevertheless, studies on the application of this class of molecules for tin metal are still limited. Among the existing physiological amino acids, proline (Pro, Fig. 1) is a cyclic molecule with two possible protonation sites (*i.e.*, –COOH and –NH_3_^+^).^21^ The potential benefit of Pro against tin degradation in saline environments has not yet been studied. Thus, we conducted an extensive research with Pro amino acid towards the inhibition of tin corrosion.

Nowadays, computational studies, such as the *in silico* approach, have been mostly applied to explain observed inhibition behaviors.^22,23^ Specifically, the quantum chemical and molecular mechanic calculations have been used.^24,25^ The key assumption of this approach is the observed inhibition is a result of the interfacial interactions between the inhibitor molecules and protected metal surface. On the other hand, it is well known that the capability of an inhibitor compound is related to many factors such as its molecular structure, its concentration, metal, pH and temperature. For instance, it was reported that the pH of the solution plays a decisive role, in which an inhibitor can be effective at certain pH values, while being nominal at other values.^16^ Specifically, a variation in pH leads to substantial modifications in the inhibition system, especially the protonation state of the inhibitor molecule and the chemical composition of the metal surface. This means that the inhibitor/metal interactions will be affected by this variation, and subsequently, the observed inhibition behavior will also be modified. Considering these facts, by applying the *in silico* approach, the role of pH on the Pro/tin interfacial interactions was investigated in this study.

According to the views mentioned earlier, we aimed to understand the effect of pH on the inhibition process of Pro toward tin corrosion, which was limited here to acidic and near-neutral pH values (*i.e.*, 2 and 5). Initially, DFT-based calculations and Monte Carlo simulations were used to study the influence of the solution pH on the interfacial Pro/tin interactions, and then predict the inhibition trend of Pro. Subsequently, electrochemical techniques were employed to validate the predicted behaviors.

## 2 Methods and materials

### 2.1. Computational details

Before presenting the computational details, it is interesting to discuss the effect of the solution pH on the main components of our inhibition system, which are proline (Pro) molecules and the tin surface. Pro is a cyclic amino acid that contains two protonated groups, *i.e.*, carboxyl (–COOH) and amine (–NH_2_) functional groups. Depending on the acidity level of the media, Pro molecules can exist in different protonated-deprotonated forms according to its acidity constants of p*K*_a–COOH_ = 1.95 and p*K*_a–NH_2__ = 10.60.^26,27^ In this work, we limited our study to acid and near-neutral pH values of 2 and 5, respectively. Consequently, in acidic solution, two Pro forms could be found, which are fully (FP, ≈53%) and partially protonated (PP, ≈47%) forms (Fig. 1). However, at near-neutral pH, Pro is present in solution mainly in one form, *i.e.* the PP form (≈100%). Considering the effect of solution pH on the chemical nature of the surface of tin, it is well known that at acidic pH, it is clean and consists of pure tin (Sn), while at near-neutral pH, it is covered by stable tin oxide (SnO_2_).^28,29^ Based on these considerations, the adopted systems during the theoretical inspection were Pro(FP + PP)/Sn and Pro(PP)/SnO_2_ at acidic and near-neutral pH, respectively.

Before explicitly studying the interaction of this amino acid with the metal surface, the reactivity of the its considered Pro and its implicit interaction with the metal surface was investigated using the DFT/B3LYP method.^13^ The 6-311+G(d) basis set^30^ was used to carry out these calculations in aqueous phase employing the IEFPCM solvation model.^31^ The DFT calculations and corresponding graphic visualizations were performed using the Gaussian v.9 and GaussView v.5 software, respectively. Using the NBO v.3 program, the natural atomic charges were calculated based on the natural population analysis of the natural bond orbital.^32^ The relevant molecular electronic structure parameters‡‡The meaning of each parameter is defined in the abbreviations list., namely *E*_HOMO_, *E*_LUMO_, Δ*E*, *η*, *χ*, *μ*, TNC and Δ*N*§§To calculate Δ*N*, we used *ϕ*_Sn_ = 4.42 eV and *ϕ*_SnO_2__ = 4.75 eV., were computed as described elsewhere.^33^ The solvation free energy (Δ*G*_solv_) was also calculated.

The explicit Pro/tin interactions were evaluated utilizing Monte Carlo simulations using the COMPASS force field and considering periodic boundary conditions.^34,35^ To select a more appropriate Miller (*hkl*) surface of Sn and SnO_2_ crystals, Bravais–Friedel–Donnay–Harker analysis was conducted. Accordingly, the (111) and (110) planes were used to model the Sn and SnO_2_ substrates, respectively. Therefore, the simulations were carried out in a (13.77 × 13.77 × 28.43 Å) and (12.75 × 13.40 × 26.70 Å) simulation box for the Sn and SnO_2_ substrates, respectively, which comprised three-layers with a 20 Å vacuum thickness. Van der Waals and electrostatic non-bonding interactions were quantified using the Ewald and atom-based summation methods. All these calculations were performed in the aqueous phase using the Material Studio v.6 software. The adsorption energy of Pro on the surface of tin was calculated using eqn (1), where *E*_Sys_, *E*_Tin surface_, *E*_Pro_ and *E*_Sol_ denote the energies of the considered system, tin surface, Pro molecule form and solution, respectively.

1 *E*_ads_ = *E*_Sys_ − (*E*_Tin surface_ + *E*_Pro_) + *E*_Sol_

### 2.2. Experimental details

The electrochemical tests were conducted employing a potentiostat/galvanostat instrument (VoltaLab-PGZ301) with a three-electrode cell. Platinum, saturated calomel electrode (SCE) and pure tin metal (0.1 cm^2^) were used as the counter, reference and working electrode, respectively. Prior to each electrochemical measurement, the electrode surface was abraded using different grades of emery paper (ranging from 800 to 1200), washed with distilled water and then degreased with acetone. With the aim to reach a steady-state, the tin electrode was immersed in the examined solution for 20 min prior to further measurements. The potentiodynamic polarization (PDP) of tin was carried out at the scan rate of 1 mV s^−1^ (ref. 36–39) in the potential range of −700 to −400 mV per SCE at acid pH, whereas in near-neutral pH, potential range was −800 to −300 mV per SCE. The electrochemical impedance spectrum of tin was recorded at the potential of the open circuit in the frequency range of 3 × 10^−2^ to 10^5^ Hz with an alternative potential perturbation of ±10 mV. In the current work, all these electrochemical experiments were performed at 25 °C in stagnant 2% NaCl solutions without and with 0.005 M of Pro. The inhibition efficiency (IE) of the studied amino acid at different pH values was calculated using eqn (2) and (3) for the PDP and electrochemical impedance spectroscopy (EIS) techniques, respectively. More details on the used experimental approach can be found in our previous work.^40^2
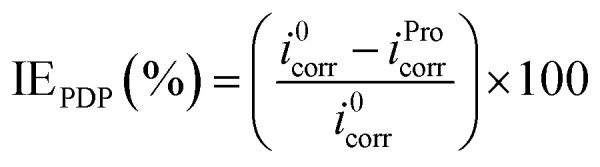
where *i*^0^_corr_ and *i*^Pro^_corr_ represent the densities of the corrosion currents of tin in the absence and presence of Pro, respectively.3
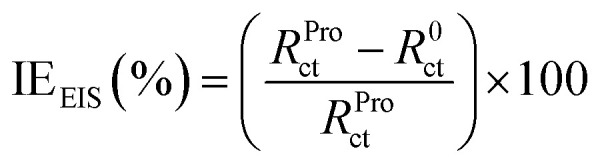
where *R*^0^_ct_ and *R*^Pro^_ct_ denote the charges transfer resistances of tin in the absence and presence of Pro, respectively.

## 3 Results and discussion

### 3.1. *In silico* anticipation

Herein, the computational approach was used to predict the effect of solution pH on the Pro/tin interactions, and therefore, the inhibition effectiveness of Pro against tin corrosion. Initially, we analyzed individual reactivity of Pro in its FP and PP forms and their implicit interactions with metal surfaces (Sn and SnO_2_) based on DFT calculations. Subsequently, in the second stage of this *in silico* inspection, the Pro/tin explicit interactions were examined employing Monte Carlo simulations.

**Fig. 2:**
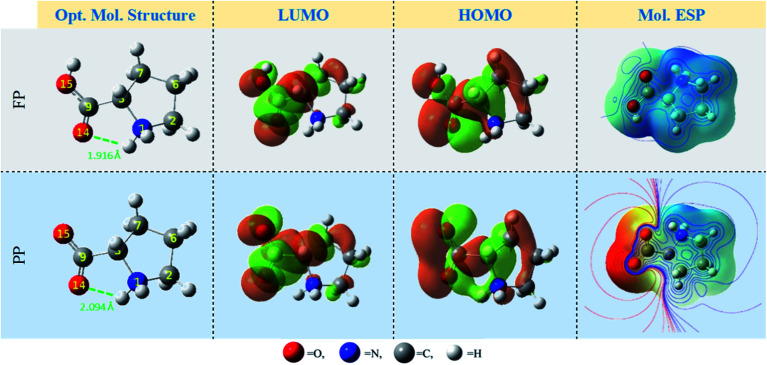
Optimized molecular structures of the Pro(FP) and Pro(PP) forms and their corresponding frontier molecular orbitals and molecular ESP contour/isosurface repartitions (regions of negative and positive ESP are red and blue colors, respectively).

**Fig. 3:**
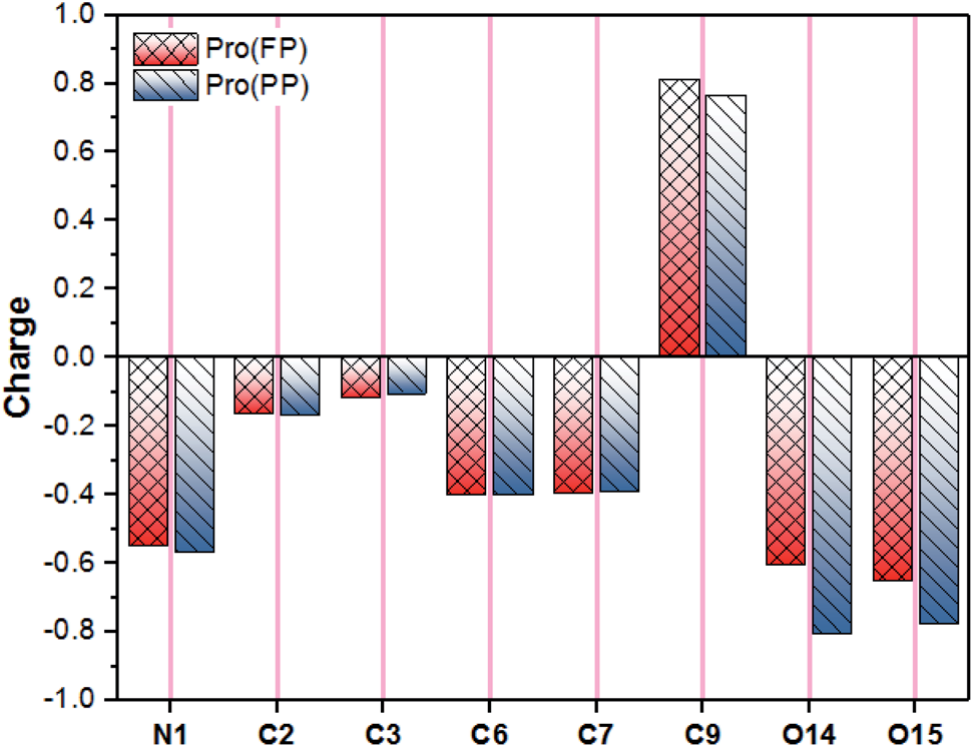
Atomic natural charges of Pro amino acid in its FP and PP forms (for atomic numbering, see Fig. 2).

Fig. 2, Table 1 and Fig. 3 present the relevant DFT results for the Pro molecule in both its considered forms in the aqueous phase. As can be seen in Fig. 2, the optimized molecule structures for both Pro forms are characterized by the presence of N–H⋯O intra-hydrogen bonds (green dashed line in Fig. 2). The calculated H-bonds lengths (1.916 and 2.094 Å) reveal the formation of strong H-bonds within the Pro molecules.^41^

Referring to Fukui's theory on frontier molecular orbitals, a molecule with the highest *E*_HOMO_ and lowest *E*_LUMO_, and consequently lowest energy gap (Δ*E*) tends to react more compared to another with a different trend.^42^ Considering our results, the Pro(PP) form shows an accurate tendency to interact with the metal surface compared to the Pro(FP) form. Moreover, following the densities of frontier molecular orbitals depicted in Fig. 2, almost of these densities are spread on the overall Pro skeleton except for the protonated amine group (shows very slight density). This particular distribution reflects that these regions are favorable sites of chemical reactivity. Also, it can be noted that the carboxyl group exhibits a remarkable density compared to the rest of the Pro molecule, especially for Pro(PP). The latter observation outlines the chemical activity of the deprotonated carboxyl group of Pro(PP) to interact with the surface of tin.

Furthermore, the hard and soft acid and base (HSAB) theory is widely used to investigate the reactivity of inhibitor molecules, which suggests that the softest molecule interacts well with a soft one and *vice versa*.^43^ Accordingly, a lower chemical hardness value (*η* = 3.351 eV) was noted for the Pro(PP) form, which indicates its improved interaction with the tin substrate, and thus is a soft material compared to the Pro(FP) form. Furthermore, based on the HSAB theory, the electronegativity (*χ*) can also be used to predict or to explain the molecular reactivity.^44^ As can be seen in Table 1, the electronegativity decreases from 5.043 eV for Pro(FP) to 3.538 eV for Pro(PP), which indicates the superior ability of the Pro(PP) form to donate electrons into the metal surface in comparison with Pro(FP) form. According to this individual analysis of the electronic structure of the considered Pro molecule forms, we can conclude that the Pro(PP) form has greater affinity toward the metal surface than the Pro(FP) form.

**Table 1:** Relevant molecular electronic structure parameters and solvation free energy of the Pro(FP) and Pro(PP) molecules using the DFT/B3LYP/6-311+G(d) level of theory under solvated conditions (IEFPCM model)

Pro form	*E*_HOMO_ (eV)	*E*_LUMO_ (eV)	Δ*E* (eV)	*η* (eV)	*χ* (eV)	*μ* (D)	TNC (e)	Δ*G*_solv_ (kcal mol^−1^)	Δ*N*_Sn_	Δ*N*_SnO_2__
FP	−8.932	−1.153	7.779	3.890	5.043	7.701	−2.882	−74.72	−0.080	—
PP	−6.889	−0.187	6.701	3.351	3.538	14.219	−3.214	−36.05	0.132	0.181

In addition to the discussed bonding interactions, the non-bonding ones play a major role in the inhibition process.^6^ To evaluate this, we used the dipole moment (*μ*) and total negative charges (TNC) of the studied molecules as descriptors (Table 1). The obtained results reveal that the higher *μ* of 14.219 D corresponds to the Pro(PP) from, while 7.701 D is found for Pro(FP). This finding is obvious due to the presence of positive (protonated amine group) and negative (deprotonated carboxyl group) net charges in the Pro(PP) molecule instead of one charge in the case of Pro(FP). According to the *μ* values, it can be anticipated that the Pro(PP) molecule can electrostatically interact better with the tin surface than the Pro(FP) molecule.^37^ Besides, a more negative TNC value was obtained for Pro(PP) compared to Pro(FP). Therefore, the Pro(PP) molecule can interact effectively through electrostatic interactions with positively charged sites on the surface of tin.^45^ The probable sites by which Pro can electrostatically interact were elucidated through the molecular electrostatic potential (ESP) map and atomic charges,^46^ which are presented in Fig. 2 and 3, respectively. It is clear from Fig. 2 that the protonated state of Pro caused by a change in pH has influenced the repartition of the ESP. The molecular skeleton of the cationic form (*i.e.*, Pro(FP)) is characterized by a positive potential (*i.e.*, electron-poor region), while a slightly negative potential (*i.e.*, electron-rich region) was observed on the protonated carboxyl group. The latter repartition changed for the Pro(PP) molecule, in which a significant negative potential is located on the deprotonated carboxyl function and the rest of the molecule is characterized by a positive potential. Similarly, in Fig. 3, an enhancement in the negative charge on the atoms of Pro(PP), in particular the O14 and O15 atoms, is perceived by the deprotonation process. Subsequently, the Pro molecule can interact electrostatically with the electropositive metallic atoms on the tin surface *via* the deprotonated carboxyl group of Pro(PP) rather than the protonated group in the Pro(FP) molecule.

Another interesting parameter the should also be considered to expect the ability of Pro to interact with the metal surface is the solvation free energy (Δ*G*_solv_). Prior to an inhibitor molecule interacting with the metallic surface, it should be released in part from its solvation shell. Consequently, the weak hydration of an inhibitor can increase its affinity to interact with a metal. According to the calculated Δ*G*_solv_ values (Table 1), a low value of −74.72 kcal mol^−1^ was found for Pro(FP), whereas a high one (−36.05 kcal mol^−1^) is noted in the case of Pro(PP). This means that Pro(FP) has a superior affinity to interact with solution components than the metal surface, while the reverse behavior is anticipated for Pro(PP). This suggests the tendency of Pro to act as a better inhibitor in a near-neutral solution in comparison with an acidic solution.

To examine the implicit connection between the Pro molecule and tin metal, the fraction of electron transfer (Δ*N*) parameter was calculated and tabulated in Table 1. It is well known that Δ*N* > 0 characterizes the tendency of a molecule to donate electrons to the metal surface, while the opposite tendency is proposed if Δ*N* < 0.^6^ As discussed in Section 2.1, at a lower pH, the Pro(FP)/Sn and Pro(PP)/Sn systems are considered. In this context, it can be noted from Table 1 that the Pro(FP) form can gain electrons from Sn surface *via* the chemical bonding process, while the opposite behavior is noted for Pro(PP) form. Moreover, the magnitude of Δ*N*_Sn_ for the Pro(PP) form is higher than that for the Pro(FP) form, which leads to a higher tendency of Pro(PP) to interact chemically with the Sn surface compared to Pro(FP). Thus, what happens at near-neutral pH when the Pro(PP)/SnO_2_ system is adopted was studied. A value of 0.181 was found, implying the transfer of electrons from the Pro molecule in its PP form to the surface of SnO_2_*via* the bonding interaction mode. Additionally, this value is the highest among the obtained |Δ*N*| values, which indicates the great capacity of the Pro(PP) molecule to interact with the tin surface at near-neutral pH.

At this stage of the current *in silico* prediction, the analysis of the electronic and non-electronic properties of Pro and its implicit interaction with the tin surface revealed the effect of the solution pH on the Pro/tin interfacial interactions in the corrosion inhibiting system. The screened amino acid is expected to show better inhibition activity for the tin metal at near-neutral pH than an acidic pH because the Pro/tin interactions at near-neutral pH are more intense than at acidic pH.

**Fig. 4:**
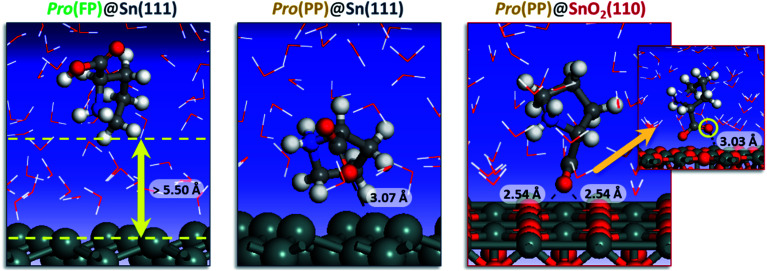
Most stable interaction configurations of the Pro(FP)@Sn(111), Pro(PP)@Sn(111) and Pro(PP)@SnO_2_(110) systems. Close interfacial Pro-surface distances are also provided.

To get detailed insights into the effect of pH on the inhibition of tin corrosion by Pro, a detailed adsorbate/substrate interaction investigation was performed using Monte Carlo simulations. The simulation was carried out for the Pro(FP)@Sn(111) and Pro(PP)@Sn(111) systems, which describe the acidic condition, while in near-neutral one, the Pro(PP)@SnO_2_(110) system was considered. Fig. 4 displays the most stable interaction configurations of these systems.

It is clear from Fig. 4 that Pro(FP) molecule does not exhibit affinity for the Sn(111) surface, in which the closest Pro(FP)–Sn(111) distance is greater than 5.50 Å. Similar behavior has been recently reported for the fully protonated cysteine form on the Fe(110) surface.^7^ Additionally, the functional groups are oriented toward the bulk solution, meaning the Pro(FP) molecule can interact favorably with the solution rather than the Sn(111) surface. This is consistent with the calculated solvation free energy previously discussed. Nonetheless, the opposite tendency was observed for the Pro(PP)@Sn(111) system, in which the Pro(PP) molecule was placed close to the Sn(111) surface. A closer interfacial distance of 3.07 Å was obtained for an oxygen atom of the deprotonated carboxyl group, whereas the other atom and protonated amine group are directly outside the Sn(111) surface. Thus, among the existing Pro forms in acidic pH, the PP form can interact more favorably with the tin surface compared to the FP form. Next, we investigated what occurs in near-neutral media, *i.e.* the Pro(PP)@SnO_2_(110) system. It is evident from Fig. 4 that the Pro(PP) molecule is closely placed on the SnO_2_(110) surface. In this interaction configuration, both oxygen atoms of the deprotonated carboxyl group are oriented toward the tin atoms on the SnO_2_(110) surface with closer interfacial distances of 2.54 and 3.03 Å. These findings reveal the strong electrostatic interaction between the negatively charged oxygen atoms of Pro(PP) and positively charged tin atoms of the SnO_2_(110) surface. Also, this proves the effective adsorption of the Pro(PP) molecule on the SnO_2_ surface *via* the deprotonated carboxyl group.

The adsorption energy was calculated to be −89.113 and −120.753 kcal mol^−1^ for the Pro(PP)@Sn(111) and Pro(PP)@SnO_2_(110) systems, respectively. These values indicate the spontaneous behaviors of the adsorption process of the Pro(PP) molecule, and its significant ability to adsorb on the SnO_2_(110) surface rather than the Sn(111) surface. Consequently, the studied amino acid can form an effective adsorbed film (*i.e.*, protective film) on the tin surface at a higher solution pH as compared to a lower pH.

Thus, all the above *in silico* results outline that a variation in solution pH can significantly affect the Pro/tin interactions, and subsequently, the observed inhibition effectiveness. In this regard, this variation can influence the protonated state of the Pro molecule and the chemical composition of the tin surface, which are considered herein. Specifically, a change in pH involves changing the overall inhibiting system. One of the key outcomes of this computational study is the prediction that the examined amino acid shows better inhibition efficiency for tin metal in near-neutral media than in acidic media.

### 3.2. Experimental verification

With the aim to validate the *in silico* predictions on the effect of solution pH on the Pro/tin interfacial interactions, and consequently the inhibition efficiency of Pro, electrochemical measurements (PDP and EIS) were performed. As stated in Section 2, 2% NaCl solution at pH 2 and 5 was chosen to represent the acid and near-neutral media, respectively, and 0.005 M concentration of Pro was used.

**Fig. 5:**
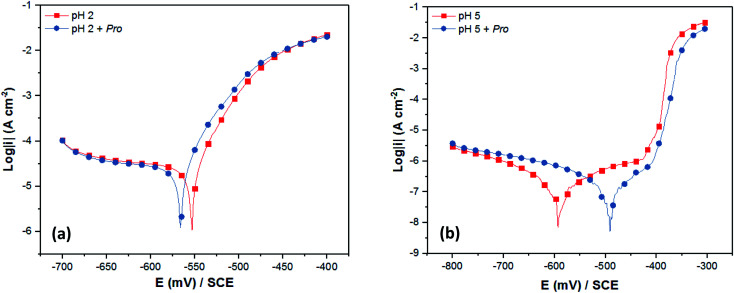
PDP plots of tin immersed in 2% NaCl solution at (a) pH 2 and (b) pH 5 without and with Pro.

Initially, the experimental verification of the anticipated trend was studied using the PDP technique. Fig. 5 presents the recorded PDP plots for tin in 2% NaCl solution in the absence and the presence of Pro at pH 2 and 5. The corresponding PDP parameters are listed in Table 2. It is clear from Fig. 5 that the solution pH noticeably influenced the shape of obtained PDP plots. At pH 2 (Fig. 5(a)), diffusion and charge transfer processes were found to control the cathodic and anodic evolutions, respectively. In contrast, at pH 5 (Fig. 5(b)), the anodic region was characterized by the formation of a thin passive film of SnO_2_ on the tin surface and the cathodic evolution was controlled by charge transfer.^47–50^ A detailed discussion on the effect of the solution pH on the corrosion behavior of tin is reported in ref. 51.

**Table 2:** PDP-derived parameters of tin in 2% NaCl solution at pH 2 and 5 without and with Pro, and calculated inhibition efficiency (IE_PDP_)

Medium	*E*_corr_ (mV)/SCE	*i*_corr_ (μA cm^−2^)	*β* a (mV dec^−1^)	IE_PDP_ (%)
pH 2	−553	25.59	32.51	—
pH 2 + Pro	−566	21.54	33.25	16
pH 5	−591	0.29	−207.71	—
pH 5 + Pro	−491	0.10	−115.02	65

a *β*_a_ at pH 2 and *β*_c_ at pH 5.

On the other hand, the addition of Pro to the corrosive solutions did not modify the overall PDP profile, which indicates the conservation of the corrosion mechanism of tin in the presence of Pro for both pH values. According to the corrosion potential (*E*_corr_) values, Pro can block both the anodic and cathodic sites on the tin surface in acid media, in which the shift in *E*_corr_ is lower than 85 mV with respect to the blank solution.^52^ However, significant potential shifting toward anodic values was observed in the near-neutral solution by adding Pro, which highlights its anodic character. As can also be seen in Table 2, the corrosion current density (*i*_corr_) of tin decreased in the presence of Pro at both pH values with respect to the uninhibited solutions, which reveals the retardation of tin corrosion by adding this amino acid. In terms of the inhibition efficiency (IE_PDP_) of Pro, the obtained value at pH 5 (65%) is higher than that at pH 2 (16%). This means that Pro acts as a better inhibitor for tin metal in near-neutral solution compared to acidic solution, which is in a good agreement with our *in silico* prediction.

**Fig. 6:**
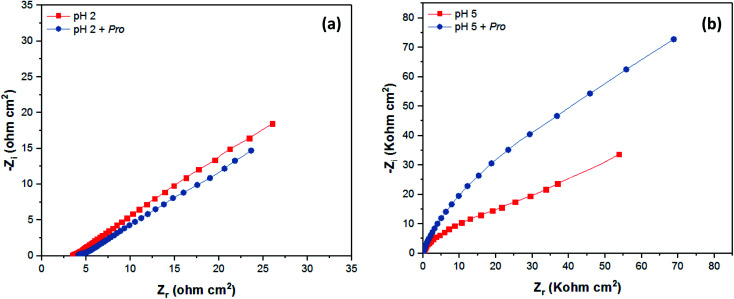
EIS data as Nyquist plots of tin immersed in 2% NaCl solution at (a) pH 2 and (b) pH 5 without and with Pro.

The corrosion behavior of tin in 2% NaCl solution without and with the addition of Pro at pH 2 and 5 was also studied employing the EIS technique. The EIS results are presented graphically as Nyquist plots in Fig. 6, while the Bode diagrams are provided in the ESI (Fig. S1†). The recorded spectra reveal the considerable effect of pH on the mechanism of tin corrosion, as highlighted using the PDP technique. In acidic solution (Fig. 6(a)), the EIS spectrum purely demonstrated a straight line, corresponding to the Warburg impedance (*W*), which indicates the diffusion of the solution components at the tin/solution interface.^53^ However, in near-neutral solution (Fig. S1†), the broad character of the phase angle plots clearly indicates the presence of two time constants.^54^ According to Fig. 6(b)), a depressed semicircle can be observed in the high-frequency region followed by a Warburg line in the low-frequency region.^51^ Moreover, even in the presence of Pro, the spectra of tin remain similar to that in its absence for both pH values. These observations confirm the PDP results, where the solution pH significantly influences the mechanism of tin corrosion, whereas the presence of Pro does not.

**Fig. 7:**
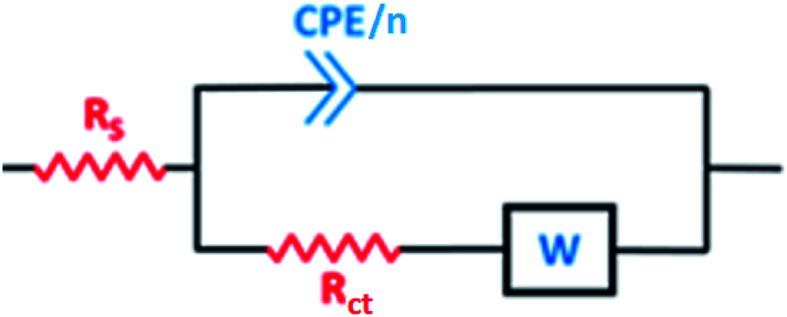
EEC model used to fit the data in Fig. 6(b).

As shown in the spectra at pH 2, only Warburg impedance can be observed (Fig. 6(a)), and thus we limit the discussion to the EIS-derived parameters at pH 5 only, as adopted elsewhere,^40^ whereas at pH 2 the details are provided in Table S1 (see ESI†). To fit the recorded spectra at pH 5 (Fig. 6(b)), the equivalent electrical circuit (EEC) model in Fig. 7 was utilized, as implemented in the ZSimpWin 3.10 software. In this figure, *R*_s_ represents the solution resistance, CPE/*n* is the constant phase element (*n* denotes the surface irregularity index), which is used to describe the inhomogeneity of the metal surface, ‘*R*_ct_’ is the charge transfer resistance and ‘*W*’ is the Warburg's impedance. The calculation of ‘CPE/*n*’ was carried out using eqn (4), where ‘*Q*’ is the magnitude, ‘*j*’ represents the imaginary root, ‘*ω*’ represents the angular frequency and ‘*n*’ is the exponential term.^55^ The double layer capacitance (*C*_dl_) value was calculated employing eqn (5), where ‘*f*_max_’ represents the frequency value corresponding to the maximum phase angle plot.^56^ The derived electrochemical parameters and calculated inhibition efficiency (IE_EIS_) are listed in Table 3. As shown in this table, the value of *Q* decreased with the addition of Pro, signifying its adsorption onto the tin surface, which led to the formation of a protective film on the metal surface under these conditions.^57^ Furthermore, the value of ‘*n*’ decreased in the presence of Pro, which indicates an increase in the inhomogeneity of the tin surface with the addition of this amino acid. This behavior further confirms the adsorption of Pro onto the tin surface.^58^ On the other hand, an increase in the *R*_ct_ of tin was noted by introducing Pro in the solution at pH 5, which reflects its ability to prevent tin corrosion.^59^ The calculated IE_EIS_ is 60%, which is comparable to that obtained from the PDP technique.

4 *Z*_CPE_ = [*Q*(*jω*)^*n*^]^−1^

5 *C*_dl_ = *Q*(2π*f*_max_)^*n*−1^

**Table 3:** EIS-derived parameters of tin in 2% NaCl solution at pH 5 without and with Pro and the calculated inhibition efficiency (IE_EIS_)

Solution	*R*_s_ (ohm cm^2^)	*Q* (μS s^*n*^ cm^−2^)	*n*	*C*_dl_ (μF cm^−2^)	*R*_ct_ (kohm cm^2^)	*W* (μS s^5^ cm^−2^)	IE_EIS_ (%)
pH 5	4.9	47.68	0.927	30.296	22.800	56.28	—
pH 5 + Pro	13.3	43.35	0.856	18.625	57.070	17.30	60

**Fig. 8:**
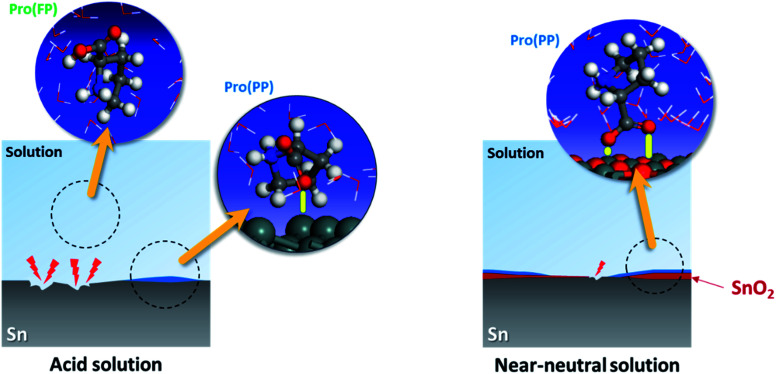
Role of solution pH on the inhibition activity of Pro toward tin corrosion.

Based on the experimental data, the studied amino acid exhibited more remarkable inhibition effectiveness for tin metal in near-neutral media than in acidic media, which validates our *in silico* prediction. These findings prove the substantial effect of the solution pH on the ability of Pro to act as a corrosion inhibitor for tin. This is attributed to the role of this factor on the Pro/tin interfacial interactions, as discussed fully in Section 3.1. Fig. 8 displays a simplified schematic of the role of pH on the inhibition process of Pro against tin corrosion.

## 4 Conclusions

The role of the solution pH on the interactions between Pro molecule and tin surface were investigated theoretically and then validated experimentally in terms of inhibition efficiency. DFT-based calculations and Monte Carlo simulations were performed for the theoretical prediction, while two common electrochemical techniques (PDP and EIS) were employed to validate these computational predictions. The analysis of the electronic and non-electronic parameters of Pro (FP and PP forms) and its implicit and explicit interactions with the metal surface (Sn and SnO_2_) demonstrated the considerable effect of the solution pH on the inhibition process. It was predicted that Pro can act as a more efficient corrosion inhibitor for tin in near-neutral solution than in acidic solution. This is attributed to the remarkable affinity of the Pro(PP) form to interact with the tin metal SnO_2_(110) surface *via* its deprotonated carboxyl group in near-neutral media. However, in acidic media, its affinity toward the Sn(111) surface is lower, whereas the Pro(FP) form was found to be solvated in solution, and thus unfavorably interacted with the Sn(111) surface. The latter anticipated behavior was confirmed experimentally, in which the inhibition effectiveness of around 65% was obtained for Pro at pH 5, whereas 16% was found at pH 2. This indicates that the inhibition capability of Pro is better in near-neutral media than in acidic media. Furthermore, this work proves the possibility to study the effect of an experimental parameter (*e.g.*, pH) on the inhibition efficiency of an inhibitor compound *via* the employed computational approach.

## Author contributions

B. E. I. conducted the theoretical/experimental studies and drafted the manuscript; L. B. and S. E. I. revised the manuscript and supervised the whole work.

## Abbreviations

Pro: Proline
FP: Fully protonated
PP: Partially protonated
IEFPCM: Integral equation formalism polarizable continuum model
DFT: Density functional theory
*E*_HOMO_
: Energy of highest occupied molecular orbital
*E*_LUMO_
: Energy of lowest unoccupied molecular orbital
Δ*E*: Gap energy
*η*
: Hardness
*χ*
: Electronegativity
*μ*
: Dipole moment
TNC: Total negative charger
Δ*N*: Fraction of electron transfer
PDP: Potentiodynamic polarization
EIS: Electrochemical impedance spectroscopy

## Conflicts of interest

There are no conflicts to declare.

## Supplementary Material

RA-010-D0RA04333H-s001
