# Supplementary material for: The role of pH in corrosion inhibition of tin using the proline amino acid: theoretical and experimental investigations
Source: RSC Adv. 2020 Aug 11;10(50):29696–704. doi: 10.1039/d0ra04333h (PMC9056173; doi:10.1039/d0ra04333h)
Supplement: RA-010-D0RA04333H-s001 [file RA-010-D0RA04333H-s001.pdf]

Electronic Supplementary Material (ESI) for RSC Advances.
This journal is © The Royal Society of Chemistry 2020

Supplementary Data

The Role of pH on the corrosion inhibition of tin using proline amino acid: Theoretical and experimental investigations

Brahim EL IBRAHIMI*[a], Lahcen BAZZI[b], Souad EL ISSAMI[a]

[a] *Applied Chemistry-Physic Team, Faculty of Sciences, University of Ibn Zohr, P.O. Box 8106, Cité Dakhla, Agadir, Morocco.*

[b] *Industrial & Logistic Laboratory, Higher School of Management, Telecommunications and Computer Science, SUP MTI, Rabat, Morocco.*

***Corresponding author**

E-mail address: brahimmhm@gmail.com - Phone number: +212672254020

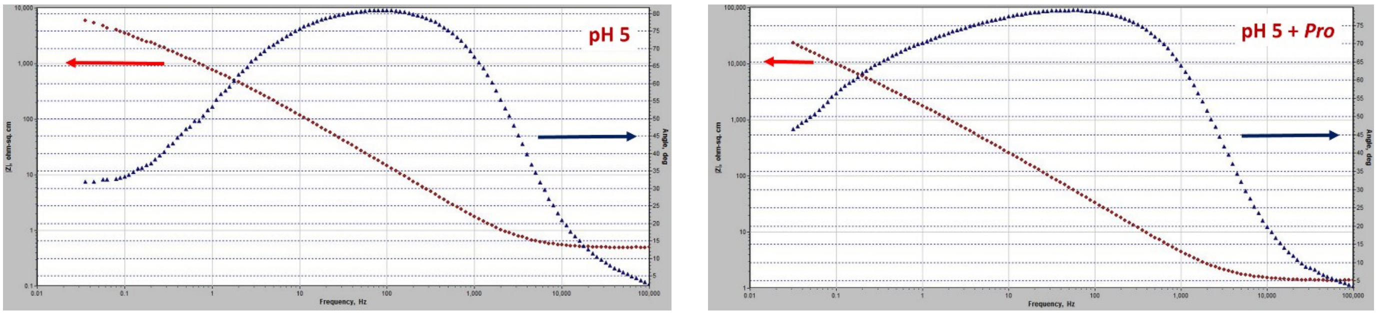

Fig. S1. Bode diagrams of tin immersed in 2% NaCl solution at pH 5 without and with *Pro*.

Table S1. EIS-derived parameters of tin in 2% NaCl solution at pH 2 without and with *Pro*

**Medium**	**R_s (ohm cm^2)**	**Q (µS s^n cm^{-2})**	**n**	**W (µS s^5 cm^{-2}) 10^{-6}**
pH 2	0.355	0.733	0.4452	0.957
pH 2 + *Pro*	0.427	0.837	0.4107	5.744
